# Visual Modulation of Resting State α Oscillations

**DOI:** 10.1523/ENEURO.0268-19.2019

**Published:** 2020-01-02

**Authors:** Kelly Webster, Tony Ro

**Affiliations:** 1 Program in Psychology; 2 Program in Biology; 3 Program in Cognitive Neuroscience, The Graduate Center of the City University of New York, New York, NY 10016

**Keywords:** α peak frequency, α power, EEG, neural oscillations

## Abstract

Once thought to simply reflect passive cortical idling, recent studies have demonstrated that α oscillations play a causal role in cognition and perception. However, whether and how cognitive or sensory processes modulate various components of the α rhythm is poorly understood. Sensory input and resting states were manipulated in human subjects while electroencephalography (EEG) activity was recorded in three conditions: eyes-open fixating on a visual stimulus, eyes-open without visual input (darkness), and eyes-closed without visual input (darkness). We show that α power and peak frequency increase when visual input is reduced compared to the eyes open, fixating condition. These results suggest that increases in α power reflect a shift from an exteroceptive to interoceptive state and that increases in peak frequency following restricted visual input (darkness) may reflect increased sampling of the external environment in order to detect stimuli. They further demonstrate how sensory information modulates α and the importance of selecting an appropriate resting condition in studies of α.

## Significance Statement

α oscillations have long been considered to reflect a stable neural trait, but we demonstrate that α varies with sensory input. By manipulating both eye state and sensory input, we demonstrate that under resting state conditions, visual input drives changes in α power and peak frequency. These changes likely allow the visual system to dynamically switch between interoceptive and exteroceptive states to prioritize detection of weak and/or infrequent visual stimuli. This work has important implications for studies of resting state brain dynamics, which have traditionally employed a variety of resting state conditions that may or may not match task conditions in terms of sensory input.

## Introduction

Neuronal oscillations have provided considerable insights into the dynamics of information processing and cognition in the brain. Of particular interest in the fields of attention and perception is the 7- to 13-Hz α oscillation. Once thought to reflect cortical idling (Adrian and Matthews, 1934), the α rhythm is now known to play an active role in cognitive processing. Specifically, α is thought to be a mechanism of functional inhibition, in which α synchronization increases over task-irrelevant areas and decreases over task-relevant ones. Such changes may increase the signal-to-noise ratio in neural processing and improve performance. Indeed, enhanced visual perception and detection are associated with reduced α power (Ergenoglu et al., 2004; Hanslmayr et al., 2007; Romei et al., 2008; Mathewson et al., 2009) and certain phases of the α cycle (Mathewson et al., 2009; Jaegle and Ro, 2014). The causal role of the α rhythm in subsequent perception and cognition has been well demonstrated (Thut et al., 2011; Jaegle and Ro, 2014), but the role of cognitive and sensory processes in modulation of the α rhythm is unclear.

Task-related changes in α power may reflect changes in attentional direction or cognitive state (Worden et al., 2000). Ray and Cole (1985a,b) demonstrated that α power increases over parietal electrodes during tasks that induce an interoceptive state (i.e., attention toward internal cognitive processes) relative to those that induce an exteroceptive state (i.e., attention toward external input). Similarly, α power increases during mental imagery compared to actual perception (Schupp et al., 1994; Cooper et al., 2003). However, changes in α power have been demonstrated in contexts where the role of attention is less clear, like during eye closure (Berger, 1929). Therefore, there may be different mechanisms for these power changes.

Studies have attempted to elucidate whether α power changes during eye closure are endogenously or exogenously driven. Adrian and Matthews (1934) demonstrated that while the α rhythm is induced most readily during eye closure, any uniform visual field can evoke α, suggesting an attentionally-driven effect. More recently, Boytsova and Danko (2010) demonstrated that eye closure modulates α power in darkness, and Ben-Simon et al. (2013) extended these findings to light conditions. Interestingly, comparing eyes-open conditions indicated that occipital α power increases in darkness compared to light. While this suggests that sensory input affects α power over posterior regions, Ben-Simon et al. (2013) focused the remainder of their interpretations on the frontal α effect and concluded that shifts in attentional direction modulate changes in α power. Therefore, the magnitude of sensory and state change effects on α power and the role of attention remain unclear.

The effects of endogenous and exogenous changes on other attributes of the α rhythm, like peak frequency, are even less understood. α peak frequency has long been considered to be a stable trait with high test-retest reliability (Gasser et al., 1985; Salinsky et al., 1991; Kondacs and Szabó, 1999; Grandy et al., 2013). However, recent evidence suggests that changes in cognitive (Haegens et al., 2014) and physical (Hülsdünker et al., 2016) demands cause shifts in α peak frequency.

The role of α peak frequency on subsequent perception has received considerable interest (Cecere et al., 2015; Samaha and Postle, 2015; Ro, 2019), and recently, studies have begun to investigate the opposite question: how sensory changes influence the α rhythm. However, these sensory effects are unclear and may be influenced by task and/or stimulus parameters. For example, some studies indicate that under task conditions, α peak frequency increases with increasing luminance of a peripheral stimulus (Cohen, 2014), while others indicate that peak frequency decreases or stays the same (Benedetto et al., 2018). Conversely, under resting conditions, α power, but not peak frequency, increases with reduced luminance (Benedetto et al., 2018). Neither study investigated conditions of complete darkness or examined the effect of eye closure. Therefore, whether and how sensory input affects the α rhythm independent of eye closure remains inconclusive.

To clarify how the α rhythm is modulated, we measured α power and peak frequency while manipulating eye state and sensory input. If α is modulated by endogenous changes, like attentional direction, power and peak frequency differences would be expected between eyes-open and eyes-closed conditions, regardless of sensory input. However, if α is modulated by sensory input, power and peak frequency differences would be expected as visual input is eliminated, such as when the eyes are closed or the lights are extinguished. Understanding how the α rhythm is modulated speaks to a broader question of what α represents and how α changes facilitate cognitive and sensory functioning.

## Materials and Methods

This research was approved by the Institutional Review Board of the City University of New York. All participants gave written informed consent before participation.

### Participants

Eighteen adults were recruited for participation in this study. Of these, two were excluded from the analyses: one because she did not complete all three experimental conditions, and one because he reported seeing some light during the eyes-open dark (EOD) condition. The remaining 16 participants (five females, mean age of 28.06 years, 21–40 years old) completed all three conditions.

### Electroencephalography (EEG) recording and analysis

EEG activity was recorded from 18 gold electrodes using Grass amplifiers (Natus Medical Inc.) at a 1000-Hz sampling rate with an online bandpass filter of 0.1–100 Hz. Scalp electrodes were placed at the following locations in the 10-20 system: F3, Fz, F4, C3, Cz, C4, P3, Pz, P4, O1, Oz, O2. Electrodes were also placed over the right and left mastoids, above and below the left eye, and lateral to the outer canthus of the right eye. The ground electrode was placed on the forehead. EEG activity was referenced online to the left mastoid and re-referenced offline to the average of the two mastoids. In each condition, data were recorded continuously for ∼5.5 min to allow for high-resolution frequency analyses of the data.

**Figure 1.**
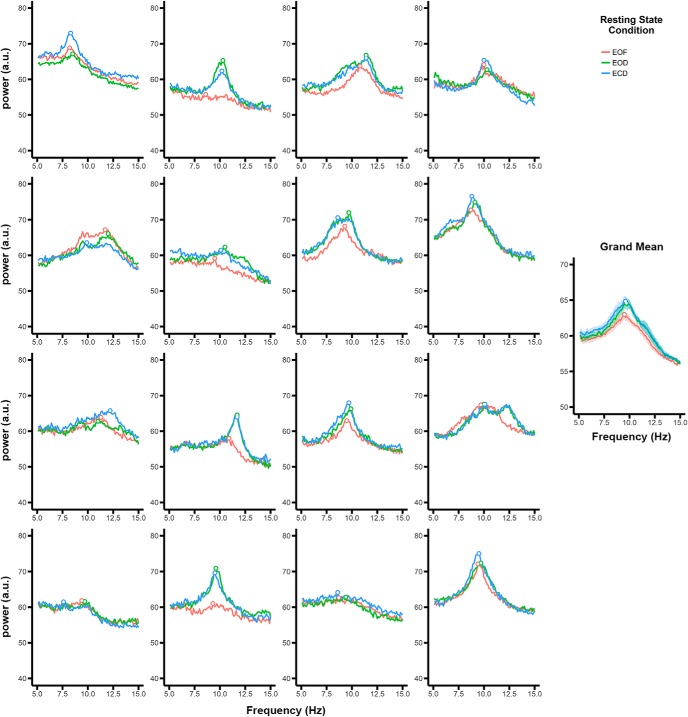
Power spectra for each subject in each of the conditions. Each panel represents data from one subject averaged across electrodes. Points represent the peak frequency for each condition. The grand mean across subjects is shown on the right. Shaded regions represent the within-subject SE (Cousineau, 2005; Morey, 2008).

The EEG data were analyzed in R with minimal pre-processing to avoid distortion of the data. Electrooculographic (EOG) artifacts originating from blinks and eye movements were removed from the raw data using ICA with the infomax ICA algorithm (Bell and Sejnowski, 1995). The independent components were visually inspected by the experimenter after the data were coded blind to condition to eliminate any potential biases. EOG components were identified through inspection of component activity, scalp maps, and power-frequency spectra. For each subject and condition, one component representing blink activity and one component representing horizontal (i.e., saccadic) activity was removed. Data were inspected after ICA removal to confirm that EOG artifacts were successfully removed. A sliding temporal window was used to epoch the data from each condition into 10-s segments with 90% overlap, resulting in 322 epochs. This epoching approach improved the reliability of the spectral estimates while maintaining high-frequency resolution. For each epoch, power spectra were computed using a fast Fourier transform (FFT) over each 10-s segment and were log transformed to reduce the 1/f effect. To analyze the overall effect of resting state condition on α peak frequency and power, the FFTs over each epoch for each condition were averaged. α power was defined as the maximum peak deflection within the 7- to 13-Hz window; the main effects of resting state condition and electrode on α power were replicated when using the mean power across the α window rather than the maximum peak inflection. An α peak could be identified for all subjects in all channels and conditions. Peak alpha frequencies for each subject in each condition can be seen in Figure 1. Because we were able to identify a peak within the α range for all subjects, α peak frequency was defined as the frequency of the maximum deflection within the 7- to 13-Hz window. This minimized the potential confounding effects of power differences on frequency extraction with a center-of-mass analysis.

### Procedure

EEG activity was recorded during three within-subjects resting conditions that varied in visual input and eye state. In the eyes-open fixation (EOF) condition, participants were instructed to fixate on the center of a screen flanked by four black fixation crosses (each subtending 0.2° by 0.2° visual angle for the horizontal and vertical components, respectively) on a gray background (luminance = 14.18 cd/m^2^). In the eyes-open dark (EOD) and eyes-closed dark (ECD) conditions, all light sources in the room were eliminated and the participant wore opaque goggles to further eliminate any light from entering the eyes. In the EOD condition, participants had their eyes open, whereas in the ECD condition, participants were instructed to close their eyes. In both dark conditions, participants were instructed to keep their eyes still, as though they were looking at an invisible fixation point at the center of their vision. Participants did not undergo dark adaptation before recording the dark conditions. EOG activity was used to confirm that participants complied with the instructions (e.g., blinks occurred during the EOF and EOD conditions and did not occur during the ECD condition and eye movements were equally minimized in all three conditions). The order of the conditions was counterbalanced across participants.

### Statistical analysis

Data were analyzed using paired *t* tests, two-way repeated measures (RM) ANOVA, and three-way RM ANOVA using R. *Post hoc* analyses for two-way and three-way RM ANOVA were performed using planned paired *t* tests and *t* tests corrected with false discover rate (FDR) for multiple comparisons. Significant differences were accepted at *p* < 0.05. For a summary of the statistical tests, see Table 1.

**Table 1.** Statistical table

	Data structure	Type of test	Power/confidence interval
a	Normal distribution	Two-way RM ANOVA	η_p_^2^ = 0.50
b	Normal distribution	*t* test (*post hoc* test)	ECD vs. EOD, mean = −31.44 (−51.38 to −11.50) ECD vs. EOF, mean = −66.31 (−94.07 to −38.56) EOD vs. EOF, mean = −34.88 (−64.18 to −5.57)
c	Normal distribution	Two-way RM ANOVA	η_p_^2^ = 0.02
d	Normal distribution	Three-way RM ANOVA	η_p_^2^ = 0.41
e	Normal distribution	Three-way RM ANOVA	η_p_^2^ = 0.35
f	Normal distribution	Three-way RM ANOVA	η_p_^2^ = 0.07
g	Normal distribution	*t* test (*post hoc* test)	EOF vs. EOD, mean = −.59 (−1.01 to −.17) EOF vs. ECD, mean = −.74 (−1.12 to −.37) EOD vs. ECD, mean = −.16 (−.30 to .12)
h	Normal distribution	*t* test with FDR correction (*post hoc* test)	P3 vs. F3, mean = .71 (.25 to 1.17) P3 vs. Fz, mean = .67 (.26 to 1.08) P3 vs. F4, mean = .63 (.24 to 1.03) P3 vs. C3, mean = .43 (.11 to .75) P3 vs. Cz, mean = .54 (.13 to .94) P3 vs. C4, mean = .63 (.28 to .99) Pz vs. F3, mean = .76 (.26 to 1.26) Pz vs. Fz, mean = .72 (.33 to 1.11) Pz vs. F4, mean = .68 (.29 to 1.07) Pz vs. C3, mean = .48 (.13 to .83) Pz vs. Cz, mean = .59 (.20 to .97) Pz vs. C4, mean = .68 (.30 to 1.07) P4 vs. F3, mean = .66 (.16 to 1.17) P4 vs. Fz, mean = .62 (.21 to 1.04) P4 vs. F4, mean = .59 (.19 to .99) P4 vs. C3, mean = .39 (−.003 to .78) P4 vs. Cz, mean = .49 (.05 to .93) P4 vs. C4, mean = .59 (.27 to .90) O1 vs. F3, mean = .81 (.34 to 1.29) O1 vs. Fz, mean = .77 (.30 to 1.23) O1 vs. F4, mean = .73 (.27 to 1.19) O1 vs. C3, mean = .53 (.14 to .91) O1 vs. Cz, mean = .63 (.17 to 1.09) O1 vs. C4, mean = .73 (.32 to 1.14) Oz vs. F3, mean = .73 (.28 to 1.17) Oz vs. Fz, mean = .69 (.24 to 1.13) Oz vs. F4, mean = .65 (.19 to 1.10) Oz vs. C3, mean = .45 (.07 to .82) Oz vs. Cz, mean = .55 (.08 to 1.02) Oz vs. C4, mean = .65 (.24 to 1.06) O2 vs. F3, mean = .74 (.25 to 1.24) O2 vs. Fz, mean = .70 (.24 to 1.16) O2 vs. F4, mean = .66 (.19 to 1.14) O2 vs. C3, mean = .46 (.08 to .85) O2 vs. Cz, mean = .57 (.09 to 1.04) O2 vs. C4, mean = .66 (.24 to 1.09) F3 vs. Fz, mean = −.04 (−.29 to .21) F3 vs. F4, mean = −.08 (−.39 to .23) F3 vs. C3, mean = −.28 (−.58 to .03) F3 vs. Cz, mean = −.17 (−.47 to .12) F3 vs. C4, mean = −.08 (−.52 to .37) Fz vs. F4, mean = −.04 (−.19 to .11) Fz vs. C3, mean = −.24 (−.54 to .07) Fz vs. Cz, mean = −.14 (−.29 to .02) Fz vs. C4, mean = −.04 (−.41 to .34) F4 vs. C3, mean = −.20 (−.53 to .13) F4 vs. Cz, mean = −.10 (−.27 to .07) F4 vs. C4, mean = 0.00 (−.29 to .29) C3 vs. Cz, mean = .10 (−.14 to .34) C3 vs. C4, mean = .20 (−.18 to .58) Cz vs. C4, mean = .10 (−.29 to .48) P3 vs. Pz, mean = −.05 (−.17 to 07) P3 vs. P4, mean = .05 (−.11 to .20) P3 vs. O1, mean = −.10 (−.24 to .04) P3 vs. Oz, mean = −.01 (−.18 to .15) P3 vs. O2, mean = −.03 (−.18 to .12) Pz vs. P4, mean = .10 (−.07 to .26) Pz vs. O1, mean = −.05 (−.26 to .17) Pz vs. Oz mean = .04 (−.21 to .28) Pz vs. O2, mean = .02 (−.19 to .23) P4 vs. O1, mean = −.14 (−.37 to .09) P4 vs. Oz, mean = −.06 (−.28 to .16) P4 vs. O2, mean = −.08 (−.27 to .11) O1 vs. Oz, mean = .08 (−.05 to .21) O1 vs. O2, mean = .06 (−.08 to .21) Oz vs. O2, mean = −.01 (−.12 to .09)
i	Normal distribution	Three-way RM ANOVA	η_p_^2^ = 0.30
j	Normal distribution	Three-way RM ANOVA	η_p_^2^ = 0.37
k	Normal distribution	Three-way RM ANOVA	η_p_^2^ = 0.10
l	Normal distribution	*t* test (*post hoc* test)	EOF vs. EOD, mean = −.02 (−.05 to .00) EOF vs. ECD, mean = −.03 (−.05 to −.01) EOD vs. ECD, mean = −.01 (−.02 to .01)
m	Normal distribution	*t* test with FDR correction (*post hoc* test)	Pz vs. F3, mean = .04 (.02 to .06) Pz vs. Fz, mean = .04 (.02 to .06) Pz vs. F4, mean = .04 (.03 to .06) Pz vs. C3, mean = .04 (.03 to .06) Pz vs. Cz, mean = .03 (.02 to .04) Pz vs. C4, mean = .04 (.03 to .05) Pz vs. P3, mean = .03 (.02 to .04) Pz vs. P4, mean = .01 (.01 to .02) Pz vs. O1, mean = .02 (.01 to .04) Pz vs. Oz, mean = .02 (.01 to .04) Pz vs. O2, mean = .01 (.00 to .03) P4 vs. F3, mean = .03 (.01 to .05) P4 vs. Fz, mean = .03 (.01 to .05) P4 vs. F4, mean = .03 (.01 to .05) P4 vs. C3, mean = .03 (.02 to .05) P4 vs. Cz, mean = .02 (.00 to .03) P4 vs. C4, mean = .03 (.02 to .04) P4 vs. O1, mean = .01 (−.01 to .03) P4 vs. Oz, mean = .01 (.00 to .03) P4 vs. O2, mean = .00 (−.01 to .02) O2 vs. F3, mean = .03 (.01 to .04) O2 vs. Fz, mean = .01 (.00 to .03) O2 vs. F4, mean = .03 (.01 to .04) O2 vs. C3, mean = .03 (.01 to .05) O2 vs. Cz, mean = .01 (.00 to .03) O2 vs. C4, mean = .03 (.01 to .04) O2 vs. P3, mean = .01 (.00 to .03) O2 vs. O1, mean = .01 (.00 to .02) O2 vs. Oz, mean = .01 (.00 to .02) F3 vs. Fz, mean = .00 (.00 to .00) F3 vs. F4, mean = .00 (.00 to .01) F3 vs. C3, mean = .01 (−.01 to .02) F3 vs. Cz, mean = −.01 (−.02 to .00) F3 vs. C4, mean = .00 (−.01 to .01) F3 vs. P3, mean = −.01 (−.03 to .00) F3 vs. O1, mean = −.02 (−.04 to .00) F3 vs. Oz, mean = −.01 (−.03 to .00) Fz vs. F4, mean = .00 (.00 to .01) Fz vs. C3, mean = .01 (−.01 to .02) Fz vs. Cz, mean = −.01 (−.02 to .00) Fz vs. C4, mean = .00 (−.01 to .01) Fz vs. P3, mean = −.01 (−.03 to .00) Fz vs. O1, mean = −.02 (−.04 to .02) Fz vs. Oz, mean = −.01 (−.03 to .00) F4 vs. C3, mean = .00 (−.01 to .02) F4 vs. Cz, mean = −.01 (−.02 to .00) F4 vs. C4, mean = .00 (−.01 to .01) F4 vs. P3, mean = −.01 (−.03 to .00) F4 vs. O1, mean = −.02 (−.04 to .00) F4 vs. Oz, mean = −.02 (−.03 to .00) C3 vs. Cz, mean = −.02 (−.02 to −.01) C3 vs. C4, mean = .00 (−.01 to .00) C3 vs. P3, mean = −.02 (−.03 to −.01) C3 vs. O1, mean = −.02 (−.04 to −.01) C3 vs. Oz, mean = −.02 (−.04 to .00) Cz vs. C4, mean = .01 (.00 to .02) Cz vs. P3, mean = .00 (−.01 to .01) Cz vs. O1, mean = −.01 (−.02 to .01) Cz vs. Oz, mean = .00 (−.02 to .01) C4 vs. P3, mean = −.01 (−.02 to .00) C4 vs. O1, mean = −.02 (−.04 to .00) C4 vs. Oz, mean = −.02 (−.03 to .00) P3 vs. O1, mean = −.01 (−.02 to .01) P3 vs. Oz, mean = .00 (−.02 to .01) O1 vs. Oz, mean = .00 (.00 to .01)
n	Normal distribution	*t* test (*post hoc* test)	Changes in sensory input over occipital electrodes versus all other electrodes, x̄ = 0.03 (0.01 to 0.04)

Table summarizes the distribution, statistical test, and power or confidence interval for each statistical test in the present study. Identifiers refer to superscript identifiers in the main text.

## Results

### Eye movements

Eye movements and blinks were measured and analyzed before their removal with ICA to ensure that participants complied with instructions. Frequent blinking during the ECD condition, for example, would suggest that participants had their eyes open, obscuring differences in α between eyes-open and closed states. Significant reductions in eye movements in the EOF condition compared to the ECD and EOD conditions could suggest that more attentional resources or cognitive control were employed to maintain fixation in the EOF condition, which would likely affect α independently of any influences of sensory input.

To compare rates of blinking across the three conditions, we counted the number of blinks occurring in each ∼5.5-min recording session before removal using ICA. These blink data were submitted to a RM ANOVA with resting state condition as a within-subjects factor and subject as a random-effects factor. There was a significant difference in the rate of blinking across the three conditions, *F*_(2,30)_ = 14.80, *p* < 0.001, η_p_^2^ = 0.50^a^. The significant *F* test was followed up by planned pairwise comparisons. Blinking was significantly reduced in the ECD (mean = 4.19, SD = 5.47) condition compared to the EOD (mean = 36.26, SD = 38.26; *t*_(15)_ = 3.36, *p* = 0.004, *d* = 0.84^b^) and EOF (mean = 70.5, SD = 52.31; *t*_(15)_ = 5.09, *p* < 0.001, *d* = 1.27^b^) conditions, confirming that participants complied with the instructions to close their eyes in the ECD condition. Blinking was also reduced in the EOD condition compared to the EOF condition, *t*_(15)_ = 2.54, *p* = 0.02, *d* = 0.63^b^.

To compare rates of eye movements across the three conditions, we counted the number of eye movements occurring in each ∼5.5 min session before removal using ICA. These eye movement data were submitted to a RM ANOVA with resting state condition as a within-subjects factor and subject as a random-effects factor. There was no significant difference in the rate of eye movements across the three conditions, *F*_(2,30)_ = 0.38, *p* = 0.69, η_p_^2^ = 0.02^c^. This suggests that eye movements were equally minimized across all three groups and that any differences in α across the three conditions cannot be explained by differences in attention or cognitive control exerted to maintain fixation.

### α peak frequency

**Figure 2.**
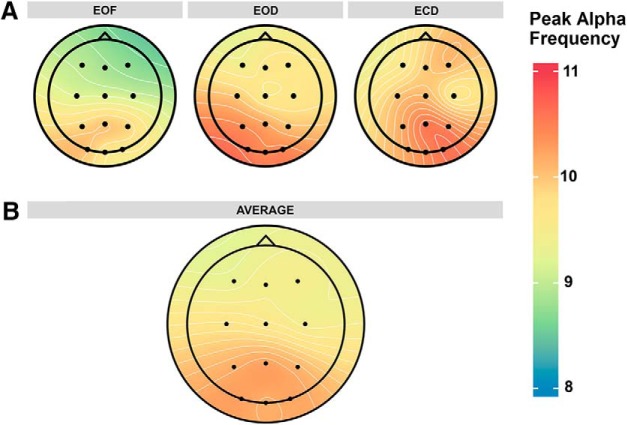
Distribution of α peak frequency across the scalp. ***A***, α peak frequency in each resting state condition. ***B***, α peak frequency averaged across resting state conditions.

α peak frequency was measured over the 12 scalp electrodes in each of the three resting state conditions (Fig. 2*A*). Before analysis of peak frequency across resting state conditions, the data were first investigated for internal consistency. This was achieved by comparing the peak α frequency data from the first half of the recording session to the second half of the recording session to measure split-half reliability. Peak frequency data were averaged across electrodes for each subject in each condition and recording half. There was high internal consistency across all three resting state conditions [*r*_EOF_(14) = 0.91, *r*_EOD_(14) = 0.98, *r*_ECD_(14) = 0.90; correlation coefficients are corrected by the Spearman-Brown formula for split-half reliability; Stanley, 1971], suggesting that our measure of peak α frequency was reliable and consistent across the recording sessions. Following analysis of internal consistency, the peak frequency data were submitted to an omnibus ANOVA with resting state condition and electrode site as within-subjects factors and subjects as a random-effects factor to compare the effects of eye closure and visual modulation on α peak frequency. There were significant main effects of resting state condition (*F*_(2,30)_ = 10.26, *p* < 0.001, η_p_^2^ = 0.41^d^) and electrode site (*F*_(11,165)_ = 8.11, *p* < 0.001, η_p_^2^ = 0.35^e^) on α peak frequency. There was no significant interaction between resting state condition and electrode site (*F*_(22,330)_ = 1.13, *p* = 0.32, η_p_^2^ = 0.07^f^).

**Figure 3.**
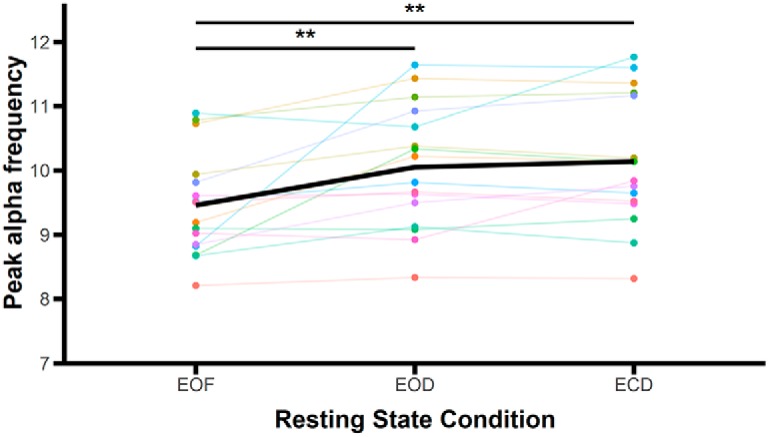
Mean α peak frequency across resting state conditions. Colored points and lines indicate data from individual subjects. The bold black line represents the mean averaged across subjects; **p < 0.01.

The significant main effects were followed up by planned paired comparisons. α peak frequency was significantly reduced in the EOF condition (mean = 9.46 Hz, SD = 0.81 Hz) compared to the EOD condition (mean = 10.05 Hz, SD = 0.96 Hz; *t*_(15)_ = 3.07, *p* = 0.008, *d* = 0.77^g^; all tests are two-tailed with α = 0.05) and the ECD condition (mean = 10.14 Hz, SD = 1.02 Hz; *t*_(15)_ = 3.72, *p* = 0.002, *d* = 0.93^g^; Fig. 3). There were no significant differences between the EOD and ECD conditions (*t*_(15)_ = 0.92, *p* = 0.37, *d* = 0.23^g^), demonstrating that the restriction of light produced these changes rather than the eyes being opened or closed.

As can be seen in Figure 2*B*, the significant effect of electrode site is driven by higher peak frequencies over posterior electrode sites compared to frontal electrode sites. Peak frequencies over posterior electrodes (P3, Pz, P4, O1, Oz, and O2) were significantly higher than peak frequencies over frontal electrodes (F3, Fz, F4, C3, Cz, and C4; all *t*s ≥ 2.52, all *p*s ≤ 0.046, all *d*s ≥ 0.63^h^, FDR corrected for multiple comparisons; with the exception of the comparisons between C3 and P4 (*t*_(15)_ = 2.12, *p* = 0.09, *d* = 0.53^h^) and Cz and P4 (*t*_(15)_ = 2.36, *p* = 0.06, *d* = 0.59^h^), which were marginally significant). There were no significant differences in peak frequency across frontal electrodes (all *t*s ≤ 1.93, all *p*s ≥ 0.13, all *d*s ≤ 0.48^h^, FDR corrected for multiple comparisons) or across posterior electrodes (all *t*s ≤ 1.46, all *p*s ≥ 0.27, all *d*s ≤ 0.36^h^, FDR corrected for multiple comparisons).

### α power

**Figure 4.**
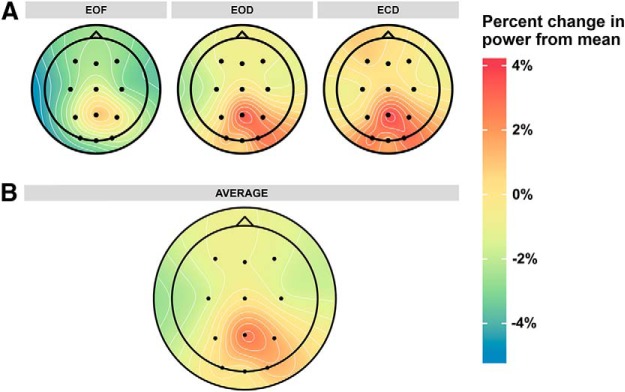
Distribution of normalized α power across the scalp. Power is plotted as percentage change from the mean across all resting state conditions, electrodes, and subjects to better illustrate the differences in power occurring across different resting state conditions. Power values that deviate strongly from the grand mean will be non-zero in the averaged data. ***A***, Normalized α power in each resting state condition. ***B***, Normalized α power averaged across resting state conditions.

α power at the α peak frequency was measured over the 12 scalp electrodes in each of the three resting state conditions. Within each subject, power values were normalized by dividing individual power values for each electrode by the mean power across all electrodes and conditions. The spatial distribution of α power across resting state conditions is shown in Figure 4*A*. Before analysis of α power across resting state conditions, the data were first investigated for internal consistency. This was achieved by comparing the α power data from the first half of the recording session to the second half of the recording session to measure split-half reliability. Power data were averaged across electrodes for each subject in each condition and recording half. There was high internal consistency across all three resting state conditions [*r*_EOF_(14) = 0.97, *r*_EOD_(14) = 0.95, *r*_ECD_(14) = 0.96; correlation coefficients are corrected by the Spearman-Brown formula for split-half reliability; Stanley, 1971], suggesting that our measure of α power was reliable and consistent across the recording sessions. Following analysis of internal consistency, normalized power values were submitted to an omnibus ANOVA with resting state condition and electrode as within-subjects factors and subjects as a random-effects factor. There were significant main effects of resting state condition (*F*_(2,30)_ = 6.51, *p* = 0.004, η_p_^2^ = 0.30^i^) and electrode site (*F*_(11,165)_ = 8.82, *p* < 0.001, η_p_^2^ = 0.37^j^) on normalized α power. There was also a significant interaction between resting state condition and electrode site (*F*_(22,330)_ = 1.84, *p* = 0.01, η_p_^2^ = 0.10^k^).

**Figure 5.**
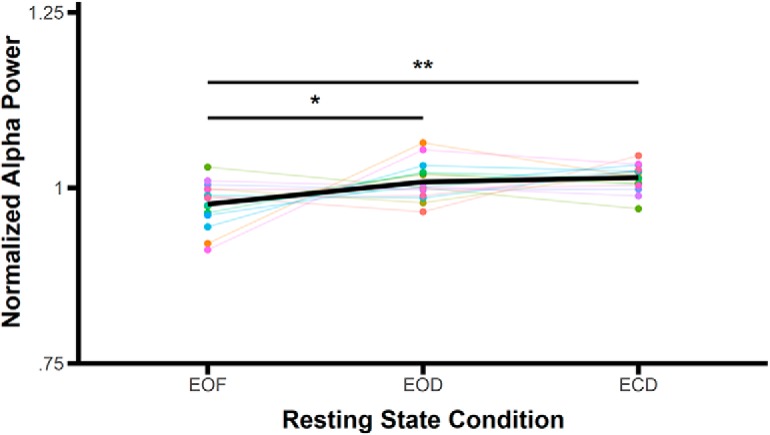
Mean normalized α power across resting state conditions. Colored points and lines indicate data from individual subjects; the same colors represent the same subjects as Figure 3. The bold black line represents the mean averaged across subjects; *p < 0.05, **p < 0.01.

The significant main effects and interactions were followed up by planned paired comparisons. α power was significantly reduced in the EOF condition (mean = 0.98, SD = 0.03) compared to the EOD (mean = 1.01, SD = 0.03; *t*_(15)_ = 2.32, *p* = 0.03, *d* = 0.58^l^) and ECD conditions (mean = 1.01, SD = 0.02; *t*_(15)_ = 3.38, *p* = 0.004, *d* = 0.85^l^; Fig. 5). There was no significant difference between EOD and ECD conditions (*t*_(15)_ = 0.75, *p* = 0.47, *d* = 0.19^l^), suggesting that changes in α power, like frequency, were modulated by sensory input, not eye closure.

As can be seen in Figure 4*B*, the significant effect of electrode site is largely driven by higher power over the posterior electrodes, in particular, Pz, P4, and O2, compared to other electrodes. α power was significantly higher in electrode Pz compared to all other electrodes except for O2 (all *t*s ≥ 3.04, all *p*s ≤ 0.027, all *d*s ≥ 0.76^m^, FDR corrected for multiple comparisons). Additionally, power over P4 and O2 was significantly higher than over most of the frontal electrodes (F3, Fz, F4, C3, and C4; power over O2 was also significantly higher than electrode Oz and power over P4 was also significantly higher than electrode P3, all *t*s ≥ 2.73, all *p*s ≤ 0.04, all *d*s ≥ 0.68^m^, FDR corrected for multiple comparisons). Power over several of the frontal electrodes was also significantly reduced relative to some of the other electrodes (power over F4 was significantly reduced compared to Cz (*t*_(df)_ = 2.73, *p* = 0.04, *d* = 0.68^m^, FDR corrected for multiple comparisons), power over C3 was significantly reduced compared to Cz, P3, O1, and Oz (all *t*s ≥ 2.76, all *p*s ≤ 0.04, all *d*s ≥ 0.69^m^, FDR corrected for multiple comparisons), and power over C4 was significantly reduced compared to Cz (*t*_(df)_ = 2.65, *p* = 0.04, *d* = 0.66^m^, FDR corrected for multiple comparisons).

To clarify the interaction between condition and electrode on α power, we examined how power changed across resting state conditions over each electrode. These changes are summarized in Table 2. As can be seen, restriction of visual input (EOD and ECD conditions compared to EOF condition) more strongly affects α power over occipital electrodes than over other electrodes. To confirm this, we tested differences in α power in light and dark conditions over occipital electrodes compared to the remaining electrodes. First, power in the dark conditions (EOD and ECD) were averaged per electrode per subject, as we found no power differences between these conditions. We subtracted the α power in the light condition (EOF) from the average dark condition per electrode per subject to get a difference score that represented changes in power as a result of light restriction. Then, for each subject, we averaged the occipital electrodes separately from the remaining electrodes. Changes in sensory input had a significantly greater effect on α power over occipital electrodes compared to the other electrodes, *t*_(15)_ = 2.87, *p* = 0.01, *d* = 0.72^n^.

**Table 2.** Percent changes in power across resting state conditions

Electrode	EOD-EOF	ECD-EOF	ECD-EOD
F3	2.62%	3.65%	1.05%
Fz	2.30%	3.28%	1.00%
F4	2.78%	3.49%	0.72%
C3	2.28%	2.95%	0.69%
Cz	1.98%	2.79%	0.83%
C4	2.43%	3.19%	0.78%
P3	2.77%	3.25%	0.50%
Pz	3.20%	3.57%	0.39%
P4	2.88%	3.26%	0.39%
O1	4.96%	5.58%	0.66%
Oz	4.15%	4.56%	0.43%
O2	5.17%	5.07%	−0.10%

The preceding analyses on the ICA-cleaned data and on data that had not been cleaned with ICA to remove eye movement and blink artifacts produced the same power and peak frequency results. Furthermore, data in the ECD condition were also analyzed after epochs with blinks artifacts were removed, which yielded the same power and frequency results. Together, these results suggest that eye movements and blinks did not significantly contaminate our data and that our ICA cleaning procedure did not distort or bias the results.

## Discussion

We measured α peak frequency and power over three different resting state conditions that varied in visual input and found that visual input modulates both α peak frequency and power. Both peak frequency and power increased in the α band when visual input was restricted. This indicates that even under resting state conditions, α peak frequency and power are variable and highly dependent on visual input. Furthermore, we demonstrate that α peak frequency and power are significantly reduced in the presence of light, but that there are no differences between dark conditions with eyes open or closed. Thus, light input, not eye closure, modulates α peak frequency and power.

Interestingly, the finding that sensory input, not eye state, drives changes in the α rhythm is somewhat at odds with other results reported in the literature. For example, in a combined fMRI and EEG study by Ben-Simon et al. (2013), eye state and electrode location, but not light input, were found to have a significant effect on α amplitude. Similarly, a resting state fMRI study by Jao et al. (2013) demonstrated that eye closure, not light input, significantly modulates the strength of functional connectivity within the default mode network, BOLD signal variance, and fractional amplitude of low frequency fluctuation within sensory cortical areas, the thalamus, and insula. The authors of both studies conclude that the brain adopts distinctive configurations when the eyes are open versus closed that are independent of sensory input and may reflect endogenous changes between interoceptive and exteroceptive states.

Although the influences of eye state and light input on resting state dynamics were investigated in both of these studies, the methodology employed in the present study differed from that employed by Ben-Simon et al. (2013) and Jao et al. (2013) in a critical way. In the present study, we used one light condition, in which eyes were open, and two dark conditions, in which eyes were open and closed. However, both the Ben-Simon et al. (2013) and Jao et al. (2013) studies employed two light (eyes open and eyes closed) and two dark (eyes open and eyes closed) conditions. While the goal of this manipulation was to fully disentangle the effects of eye closure and light input on subsequent brain dynamics, it introduces a confound: closure of the eyelids likely changes rather than eliminates the amount of illumination reaching the eyes in the light condition. This effect will vary from individual to individual based on eyelid skin thickness and pigmentation. Therefore, when averaging across the eyes open and eyes closed conditions for each lighting condition, the light condition with eyes closed likely included some visual input, which was dependent on sample characteristics, such as skin thickness and tone.

In the present study, we did not measure the α rhythm under light, eyes closed conditions, but we were nonetheless able to interpret the effect of eye closure on the α rhythm by comparing the EOD and ECD conditions, as well as the effect of sensory input by comparing the eyes open light to both the EOD and ECD conditions. However, future studies that carefully control for both illumination (sensory input) and eye state may help to further elucidate the effects of these factors on resting state brain dynamics.

Also, unlike these previous studies, our results demonstrate for the first time that visual input modulates α peak frequency under resting conditions, and that these changes in α peak frequency directly mirror power changes. While α peak frequency is typically considered to be a stable trait variable (Gasser et al., 1985; Salinsky et al., 1991; Kondacs and Szabó, 1999; Grandy et al., 2013), more recent work has demonstrated that peak frequency also functions as a state variable, with fluctuations in peak frequency potentially reflecting changes in cognitive and/or physical engagement (Haegens et al., 2014; Gutmann et al., 2015). While a few other studies have begun to investigate the effects of visual input on α peak frequency, the results heretofore are largely mixed. For example, Cohen (2014) noted that α peak frequency increased with increasing luminance of a contralateral stimulus during an infrequent time-coordinated stimulus localization task. However, a study by Benedetto et al. (2018) found the opposite effect under task conditions: when the luminance of a covertly attended disk was altered, phase-locked α peak frequency increased in low ambient luminance conditions, whereas spontaneous α was unaffected. Furthermore, ambient luminance was found to have no effect on spontaneous α frequency under resting conditions. The differences in results between these two studies and the present one may be due to several factors. Differences in the task between the Cohen (2014) and Benedetto et al. (2018) studies, especially task difficulty and task-relevance of the luminance-changing stimulus, may account for the different effects of luminance on α frequency. Furthermore, only the luminance of a relatively small square stimulus was manipulated in the Cohen (2014) study, whereas ambient luminance was also manipulated in the Benedetto et al. (2018) study, as in the present study. While ambient luminance was manipulated in both the present study and in the Benedetto et al. (2018) study, Benedetto et al. recorded EEG in mesopic luminance following dark adaptation, whereas the present study did not require any period of dark adaptation and compared mesopic and scotopic conditions; the restricted luminance range used by Benedetto et al., may therefore have masked the differences observed in the present study. Nevertheless, a more thorough examination of the interplay between task demands, stimulus characteristics, and luminance is needed to clarify the effects of visual input on α peak frequency.

What do these newfound shifts in peak frequency and power with changes in sensory input represent? Haegens et al. (2014) demonstrated that individual α peak frequency increases with increasing task demands while α power decreases. The authors interpret this shift in frequency as reflecting either a reduction in the window of suppression, which may be beneficial with an increasingly difficult task, or activation of different neuronal populations with changing task demands. Here we demonstrate increases in both peak frequency and in power with changes in sensory input, although the “task” is unchanged across conditions. A similar underlying mechanism may account for increases in α peak frequency under both conditions of reduced visual input and increased task demands. In the Haegens et al. (2014) study, α peak frequency increased during 0-back and 2-back tasks relative to resting, baseline, and passive viewing conditions, suggesting that when a task is made more difficult, reducing the window of suppression may be beneficial to improving performance. Similarly, when visual input is extinguished, as in the present study, reducing the window of suppression allows the visual system to sample the external environment more frequently to detect visual stimuli. An alternative explanation is that when visual input is reduced, the brain shifts from an exteroceptive state to interoceptive state, which may be accompanied by activation of a different population of neurons whose activity may oscillate at a higher peak frequency.

Support for this latter explanation comes from the resting state fMRI literature. Recently, there has been a growing interest in the neural processing that occurs when we “do nothing,” the so-called resting state. Eyes open and eyes closed states under varying degrees of visual stimulation have been interchangeably employed as resting conditions. Drawing from the EEG literature, however, there has been recent interest in whether these different resting conditions are actually equivalent, or if they produce distinct patterns of activity in the brain. Initially, studies determined through visual inspection that the default mode network was stable across different resting conditions (Greicius et al., 2003; Fox et al., 2005; Fransson, 2005). When compared statistically, however, studies have demonstrated that eyes open and eyes closed resting states produce different patterns of activity and connectivity in the brain. While there is some variability in the findings (possibly due to differences in data collection and processing), studies have generally demonstrated that BOLD activity is reduced under eyes open resting state conditions compared to eyes closed conditions (McAvoy et al., 2008; Bianciardi et al., 2009; Zou et al., 2009; Jao et al., 2013). Some studies have also reported decreases in functional connectivity under eyes open conditions relative to eyes closed conditions (Bianciardi et al., 2009; Zou et al., 2009; Xu et al., 2014; however, see Yan et al., 2009). Few studies have attempted to disentangle the effects of sensory stimulation from eye state on resting state activity, but one study by Jao et al. (2013) averaged across eyes open and eyes closed light conditions, which produce different illumination levels, impeding the interpretation of the effect of sensory input on subsequent activity as described above.

Different resting states have also been demonstrated to activate different systems within the brain, with eyes-open conditions producing activation in oculomotor and attentional systems and eyes closed conditions producing activation in sensory systems (Marx et al., 2003, 2004). These findings collectively suggest that eyes-open and eyes-closed resting states are two different states of cognition; eye closure induces an interoceptive state, characterized by imagination, planning, and multisensory activity, while eye opening induces an exteroceptive state characterized by attention, readiness, and oculomotor activity. The differences we see in α power and frequency in the present study may similarly reflect a state change from exteroception to interoception as visual input is restricted.

Interestingly, results from a recent study suggest that even within a single resting state condition, the brain does not remain tonically active in a single state of brain function (Fransson, 2005). Rather, there are low frequency (0.012–0.1 Hz) fluctuations in the BOLD signal that are synchronized across brain regions. Fransson (2005) demonstrated that these signal fluctuations were associated with changes in functional connectivity, such that the fluctuations in the BOLD signal represented the alternating activation of two distinct networks. This relationship between spontaneous low frequency BOLD fluctuations and functional connectivity was found in both eyes open and eyes closed resting states (although the two conditions were not compared statistically). Fransson speculated that during rest, the interoceptive state, which is characterized by inner thought and self-reflective thinking, is periodically interrupted as the brain shifts to an exteroceptive state, which is characterized by increased readiness and attention to changes in the internal and external environment. These results are interesting in light of other studies that have suggested that eyes closed resting states activate networks consistent with an interoceptive state, while eyes open resting state activate networks consistent with an exteroceptive state. Together, these results might suggest that at rest, the brain shifts from an interoceptive to exteroceptive state routinely, with signal intensity and/or functional connectivity being stronger in the interoceptive network when the eyes are closed, and vice versa. However, additional research that statistically compares eyes open and eyes closed resting states, along with resting states that differ in sensory input, during these spontaneous low frequency fluctuations will be necessary to better understand these dynamic changes in brain state at rest.

Although the fluctuations measured in the BOLD signal during rest are much slower than the α rhythm, there are nevertheless similarities between these resting state fMRI studies and the present study. We find that α power and peak frequency increase as visual input is restricted. It has been well demonstrated that perception and detection of external events is prioritized at certain phases of the α cycle. Therefore, the α rhythm itself may reflect brief fluctuations between interoceptive and exteroceptive states. Under restricted visual input, the power of this rhythm becomes higher but the frequency of these fluctuations increases, allowing the brain to sample the external environment more frequently for adaptive behavior. Additional research using combined EEG and fMRI may help to clarify the relationship between interoceptive and exteroceptive states, the α rhythm, and low frequency BOLD fluctuations.

Analyses on the rates of blinking and eye movements across resting state conditions were conducted to determine compliance with task instructions (i.e., subjects closed their eyes during the ECD condition and maintained fixation in all three conditions). We found no differences in the rate of eye movements across the three resting state conditions, indicating that eye movements were equally minimized in all three conditions. This suggests that the differences found here between light and dark conditions cannot be explained by differences in attention or cognitive control exerted to maintain fixation in the EOF condition, and instead reflect the sensory differences across conditions. We did observe differences in the rate of blinking across conditions. As would be expected, blinking was significantly reduced in the ECD condition compared to both the EOD and EOF conditions, indicating that participants complied with instructions to close their eyes. Interestingly, blinking was also significantly reduced in the EOD condition compared to the EOF condition. Rates of blinking slow with increased task demands and mental engagement, presumably to minimize the risk of missing task-relevant information during eye closure (Drew, 1951; Baumstimler and Parrot, 1971; Stern and Skelly, 1984; Oh et al., 2012). While task demands were not high during these resting state conditions, the reduced rate of blinking in the EOD condition compared to the EOF condition may again reflect a tendency of the visual system to prioritize detection of weak visual input under impoverished visual conditions, consistent with the increase in α peak frequency shown here under conditions of reduced luminance. Alternatively, the higher rates of blinking in the EOF condition compared to the EOD condition may have been a result of the light eliminating goggles used in the EOD condition, which may have produced differences in eye dryness between the two conditions.

Intraindividual shifts in α peak frequency have been noted with changes in cognitive engagement (Haegens et al., 2014), physical task demands (Hülsdünker et al., 2016), and hormone levels (Brötzner et al., 2014). Our results add to this literature and suggest that even changes in sensory input may cause shifts in α peak frequency. We demonstrate across three different resting conditions that visual input modulates both α peak frequency and power. As visual input is reduced, both α peak frequency and power increase. These results have important implications for future studies of the α rhythm. Given the impact of visual input on α peak frequency and power, care should be taken when choosing a resting comparison condition. Visual input in the resting condition should match the task condition as closely as possible, as manipulating the visual input may bias frequency and power in a way that precludes interpretation of task effects.

## Synthesis

Reviewing Editor: Bradley Postle, University of Wisconsin Decisions are customarily a result of the Reviewing Editor and the peer reviewers coming together and discussing their recommendations until a consensus is reached. When revisions are invited, a fact-based synthesis statement explaining their decision and outlining what is needed to prepare a revision will be listed below. The following reviewer(s) agreed to reveal their identity: Kyle Mathewson.

Here is the full text of the two reviews:

Reviewer #1

In their paper entitled “Visual modulation of resting state alpha oscillations” the authors report the results of an experiment in which they measure EEG during different resting state conditions. The authors report differences in power and peak frequency, not only between classic eyes open and closed conditions, but also between eyes open vs a condition where the eyes are open in the dark. The experiment seems well done. The sample size is quite small. The data analysis and representation is missing important details that make it difficult to fully evaluate. Further, this study seems like a replication of work that is over 80 years old from Adrian and Mathews 1934 paper, so the authors should do a better job of describing why the current study was needed given what we already know about how alpha is modulated in the dark with the eyes open.

To fully evaluate the suitabililty of this submission for publication, more details on the analysis and results are required.

---

Abstract:

Increase in power and frequency without visual input' - compared to what condition

shift in peak frequency = increased sampling - The authors should be clear here and throughout the manuscript on which direction and which conditoins they effects they mention relate to, it is currently quite confusing

---

Intro:

I believe the original Adrian and Mathews 1934 EEG paper has an experiment with eyes open in the dark as well, and they also discuss many of the same ideas that the current paper proposes as novel, please read the section starting with “THE FAILURE OF THE RHYTHM DURING VISUAL ACTIVITY.” - How does this current experiment advance the understanding we gained from this almost 90 year old study

In general, I feel the authors could be more clear in their languge and logic behind why this particular experiment is needed based on what is not currently known in the field, or in terms of what studies need better modern replication.

----

Methods

pg. 5 - ln 86 - “This research adhered to the tenets of the Declaration of Helsinki ” - the declaration of helsini requires all studies to be pre-registered, which this one is not, so please use other language here

pg. 6 - ln 105 - “EOG artifacts originating from blinks and eye movements were removed from the raw

106 data using ICA.” - the authors need to provide MUCH more information about this. What ICA alogirthm was used, how were components related to eye movements ID'ed, was this automatic or manual. Who did it if manual and are they well trained in this. Were there decisions made blind to the condition? How many components were removed for each participant, how was this decided. Were the data inspected before and after to see if the methods worked. I suggest the authors consider using a technique with less degrees of freedom, like the Gratton EMCP regression procedure.

pg 7, ln 114 - “An alpha peak could be identified for all subjects in all channels and 115 conditions.” - show them like Samaha and Postle

how were blinks identified if the ICA already removed them? How were they identified for this analysis, by hand or automatically? What unit is blink rate reported in on pg. 8?

“eye movement rate ” - how was this operationalized in the data for statisical comparisons?

Ill stop here at the end of the methods section in this review. I feel that these data can't be properly considered and compared without showing the acutal spectra themselves to confirm the peak and power shifts within each subject. Also the ICA and eye movement analysis need a much better description before this analysis and results can be interpreted.

---

Figures:

Figure 1 - need to show the spectra in each condition, as well as for every subject, This should be combined with much more information for the reader like the indivudla and grand average spectra, This figure is very unconvincing without the spectra itself plotted

Figure 2 - it would be helpful to show the values for each indivudal subject on this or another plot, given the low number of subjects in the study, Did all of their peak shifts occur in the same direction?

Figure 3 - if you are showing the difference from the mean for each conditoin, shouldn't the average of them zero?

Figure 4- should be combined with figure 3 along with the spectra themselves

Reviewer #2

The paper “Visual Modulation of resting alpha oscillations” investigate the effect of luminance changes in alpha expression. The authors found that resting alpha peak frequency and power were higher under scotopic condition (for both eyes open and closed) as compared to photopic condition (eyes open).

I think the results are interesting, and the current literature on alpha needs this kind of simple investigations to better understand the basic principles of alpha brain rhythms. I only have few minor comments to be addressed.

1) In figure 1 and 4 please show also single subject results as well as the power spectrum of the brain activity under the different conditions.

2) Instead of defining alpha power and peak as the maximum peak inflection and the frequency of this inflection within 7-13 Hz, you should use the mean power within this frequency band (for power) and the center of mass (for frequency). This might be important if some subjects do not show a clear alpha peak in their power spectrum.

## Author Response

Response to Reviewers

We thank the reviewers for their constructive and thoughtful comments. We have carefully considered each comment and have revised the manuscript to address their concerns in the following ways.

Reviewers' Comments:

Reviewer #1: In their paper entitled “Visual modulation of resting state alpha oscillations” the authors report the results of an experiment in which they measure EEG during different resting state conditions. The authors report differences in power and peak frequency, not only between classic eyes open and closed conditions, but also between eyes open vs a condition where the eyes are open in the dark. The experiment seems well done. The sample size is quite small. The data analysis and representation is missing important details that make it difficult to fully evaluate. Further, this study seems like a replication of work that is over 80 years old from Adrian and Mathews 1934 paper, so the authors should do a better job of describing why the current study was needed given what we already know about how alpha is modulated in the dark with the eyes open.

To fully evaluate the suitabililty of this submission for publication, more details on the analysis and results are required.

---

Abstract:

Increase in power and frequency without visual input' - compared to what condition

> The comparison condition in this sentence is the eyes open fixation condition (i.e., visual input). This has been clarified in the abstract.

shift in peak frequency = increased sampling - The authors should be clear here and throughout the manuscript on which direction and which conditoins they effects they mention relate to, it is currently quite confusing

> In this sentence, the shifts in peak frequency are associated with the two dark/no visual input conditions. This has been clarified.

---

Intro:

I believe the original Adrian and Mathews 1934 EEG paper has an experiment with eyes open in the dark as well, and they also discuss many of the same ideas that the current paper proposes as novel, please read the section starting with “THE FAILURE OF THE RHYTHM DURING VISUAL ACTIVITY.” - How does this current experiment advance the understanding we gained from this almost 90 year old study

In general, I feel the authors could be more clear in their languge and logic behind why this particular experiment is needed based on what is not currently known in the field, or in terms of what studies need better modern replication.

> Indeed, Adrian and Mathews (1934) provided valuable qualitative information about variations in alpha across different sensory and state conditions. However, their results were 1) mostly qualitative, 2) presented in terms of the presence/absence of the rhythm, and 3) on the time necessary to establish the rhythm. The current study provides a more robust examination of the magnitude of the effect of sensory and eye state changes on the alpha rhythm. We believe this investigation is especially important given that modern partial replications of the Adrian and Mathews paper have provided conflicting evidence as to the role of sensory input on alpha power (e.g., Ben-Simon et al., 2013).

More importantly, this paper's novel contribution is the replication of Adrian and Mathews' comparisons in the frequency domain. We believe the peak frequency findings, paired with the power results, have important mechanistic implications for the function of the alpha rhythm and its relationship with sensory processing. These points have been clarified in the introduction on page 4 (lines 48-50).

----

Methods

pg. 5 - ln 86 - “This research adhered to the tenets of the Declaration of Helsinki ” - the declaration of helsini requires all studies to be pre-registered, which this one is not, so please use other language here

> We have amended the language of this statement, which now reads, “This research was approved by the Institutional Review Board of the [Author University]. All participants gave written informed consent prior to participation.” (page 5, lines 84-85).

pg. 6 - ln 105 - “EOG artifacts originating from blinks and eye movements were removed from the raw

106 data using ICA.” - the authors need to provide MUCH more information about this. What ICA alogirthm was used, how were components related to eye movements ID'ed, was this automatic or manual. Who did it if manual and are they well trained in this. Were there decisions made blind to the condition? How many components were removed for each participant, how was this decided. Were the data inspected before and after to see if the methods worked. I suggest the authors consider using a technique with less degrees of freedom, like the Gratton EMCP regression procedure.

> We agree that many details about the EOG artifact removal procedure were absent from the methods section. Information describing how EOG artifacts were identified and removed has been added (page 6-7, lines 104-110). We now indicate that, “eye movements were removed from the raw data using ICA with the infomax ICA algorithm (Bell and Sejnowski, 1995). The independent components were visually inspected by the experimenter after the data were coded blind to condition to eliminate any potential biases. EOG components were identified through inspection of component activity, scalp maps, and power-frequency spectra; for each subject and condition, one component representing blink activity and one component representing horizontal (i.e., saccadic) activity was removed. Data were inspected after ICA removal to confirm that EOG artifacts were successfully removed.” We also found the same results after performing the analyses on data that had not been cleaned with ICA to remove eye movement and blink artifacts; this has been clarified in a footnote in the Methods section (page 6).

pg 7, ln 114 - “An alpha peak could be identified for all subjects in all channels and

115 conditions.” - show them like Samaha and Postle

> We now illustrate the power spectra across subjects and conditions, along with the grand mean across subjects, in Figure 1.

how were blinks identified if the ICA already removed them? How were they identified for this analysis, by hand or automatically? What unit is blink rate reported in on pg. 8?

> The analyses on blink and eye movement data were conducted prior to removal of the components by ICA; this has been clarified on pages 8-9 (lines 147-148 and 154-155). Blinks were identified through manual inspection of the data along and confirmed through inspection of the scalp maps of the independent components and through the power-frequency spectra of the components. The blink rate represents the number of blinks occurring within each ~5.5 minute recording for each resting state condition.

“eye movement rate ” - how was this operationalized in the data for statisical comparisons?

> Like the blink rate above, the eye movement rate represented the number of eye movements occurring within each ~5.5 minute recording for each resting state condition. This has been clarified on page 9 (lines 165-166).

Ill stop here at the end of the methods section in this review. I feel that these data can't be properly considered and compared without showing the acutal spectra themselves to confirm the peak and power shifts within each subject. Also the ICA and eye movement analysis need a much better description before this analysis and results can be interpreted.

> We hope the addition of the power spectra, along with the clarified details about the analysis methods, aid in the understanding and interpretation of the analyses and results.

---

Figures:

Figure 1 - need to show the spectra in each condition, as well as for every subject, This should be combined with much more information for the reader like the indivudla and grand average spectra, This figure is very unconvincing without the spectra itself plotted

> We have included individual spectra per subject and condition along with the grand mean spectra in the new Figure 1 in the revised manuscript. Figure 1 in the original submission was intended to demonstrate changes in frequency across the scalp and is included as Figure 2 in the revised manuscript.

Figure 2 - it would be helpful to show the values for each indivudal subject on this or another plot, given the low number of subjects in the study, Did all of their peak shifts occur in the same direction?

> Figure 2 (now Figure 3 in the revised manuscript) has been revised to show not only the mean across subjects, but also individual values per subject (indicated by the colored points).

Figure 3 - if you are showing the difference from the mean for each conditoin, shouldn't the average of them zero?

> (Figure 3 in the original submission is now Figure 4 in the revised submission). The data represent the difference from the mean across all conditions, channels, and subjects; this is to better show the differences in power that occur from one resting state condition to the next. As such, power values that deviate strongly from the grand mean will still be non-zero in the averaged data. This is now clarified in the figure legend for Figure 4 in the revised manuscript on page 25, lines 531-534.

Figure 4- should be combined with figure 3 along with the spectra themselves

> We have included the power spectra in the new Figure 1 in the revised manuscript. Figure 4 in the original submission is now Figure 5 in the revised manuscript and has been revised to show the individual values per subject, as suggested above.

Reviewer #2

The paper “Visual Modulation of resting alpha oscillations” investigate the effect of luminance changes in alpha expression. The authors found that resting alpha peak frequency and power were higher under scotopic condition (for both eyes open and closed) as compared to photopic condition (eyes open).

I think the results are interesting, and the current literature on alpha needs this kind of simple investigations to better understand the basic principles of alpha brain rhythms. I only have few minor comments to be addressed.

1) In figure 1 and 4 please show also single subject results as well as the power spectrum of the brain activity under the different conditions.

> We have created a new figure (Figure 1 in the revised manuscript) that shows the individual power spectra across subjects and conditions along with the grand mean across subjects. Additionally, Figures 2 and 4 of the original manuscript (now Figures 3 and 5 in the revised manuscript) show individual subject data as well as averaged data.

2) Instead of defining alpha power and peak as the maximum peak inflection and the frequency of this inflection within 7-13 Hz, you should use the mean power within this frequency band (for power) and the center of mass (for frequency). This might be important if some subjects do not show a clear alpha peak in their power spectrum.

> We were able to replicate the main effects of condition and electrode by analyzing mean power instead of power at the peak alpha frequency; we have added a footnote explaining this analysis in the Methods section. However, given that we see power differences across the different resting state conditions, we found that investigating frequency changes using center of mass/centroid frequency biased the frequency analysis. Namely, alpha frequency in lower power conditions (i.e., the EOF condition) was artificially driven towards the mean frequency (10 Hz), an effect which was minimized for the higher power conditions (EOD and ECD conditions). For this reason, and because an alpha peak could be identified for all subjects and conditions (which is now clarified in Figure 1), we analyzed frequency changes using the frequency of the maximum peak inflection within the alpha range. We have clarified the rationale behind this approach in the Methods section (page 7, lines 119-122).
